# Enhanced calibration method for wall-mounted RGSC cameras: Mitigating baseline drift caused by CT couch sagging

**DOI:** 10.1371/journal.pone.0332262

**Published:** 2025-09-29

**Authors:** Jiangtao Wang, Jinyou Hu, Xiangxiang Liu, Dashuang Luo, Yisong He, Lian Zou

**Affiliations:** Cancer Center, Sichuan Provincial People’s Hospital, University of Electronic Science and Technology of China, Chengdu, Sichuan, China; Indiana University, UNITED STATES OF AMERICA

## Abstract

**Purpose:**

The drift signal due to CT couch sagging introduces intrinsic noise into respiratory signals, impairing the accuracy of tumor motion management. The purpose of this work is to present an enhanced calibration method to reduce calibration uncertainty and mitigate baseline drift caused by table sagging for the wall-mounted Respiratory Gating for Scanner (RGSC) camera.

**Methods:**

A weight of approximately 70 kg, simulating a patient’s weight, was distributed on the CT table. The external ceiling laser light was adjusted laterally by ±10 cm to align three reflector blocks sequentially with the laser at predefined positions, ensuring accurate placement of the blocks at their corresponding positions. The blocks were then moved to the internal laser plane using the CT console. Subsequently, calibration measurements were performed at nine points at the combination of three lateral positions (the CT isocenter and ±10 cm laterally from the isocenter) and three longitudinal positions (the CT isocenter and ±15 cm longitudinally from the isocenter), by occluding the other two blocks and moving the couch longitudinally. For comparison, the Varian calibration method was also implemented.

**Results:**

The block positioning uncertainty was reduced from the millimeter level to the sub-millimeter level. For a typical 40 cm scan length of DIBH, the residual baseline drift was significantly (p-value<0.001) mitigated from 2.84 ± 0.22 mm to 0.64 ± 0.06 mm.

**Conclusion:**

The proposed calibration method provides a robust solution to minimize block positioning uncertainty and reduce baseline drift caused by CT couch sagging, enhancing the repeatability and accuracy of the wall-mounted camera calibration. Its versatility for wall- and ceiling-mounted cameras further expands its potential clinical utility.

## Introduction

Radiotherapy requires precise delivery of radiation to tumor regions to achieve optimal treatment outcomes [1–3]. However, tumors located in the chest, abdomen, and pelvis are significantly affected by respiratory movements [4–6], necessitating meticulous consideration during radiation therapy. Misestimation of tumor positions and their associated motion may result in insufficient doses of tumors and overdoses of nearby critical organs [7–9]. This issue is particularly critical in stereotactic body radiotherapy (SBRT), which employs steep dose gradients and high single-fraction doses [10–12]. A 2020 survey by AAPM Task Group 324 revealed that 95% of respondents employed motion management techniques for treating thoracic and abdominal cancers [13].

Respiratory Gating for Scanner (RGSC) [14] serves as the successor to the real-time position management (RPM) system in CT simulation. Extensive validation studies have confirmed the stability and accuracy of RGSC systems [15,16]. Recently, Varian reported that couch-mounted RGSC systems, when used with Siemens SOMATOM go and SOMATOM X scanners, exhibited baseline drift of up to 8 mm caused by table sagging [17]. Such drift can cause traces to exceed predetermined thresholds during deep inspiration breath hold (DIBH) scans or inaccurate binning in 4DCT, probably compromising the precision of radiotherapy [18,19]. Since table sagging cannot be accounted for during RGSC calibration in the couch-mounted configuration, users of these scanners must opt for either ceiling-mounted or wall-mounted camera configuration.

Varian introduced a nine-point calibration method for wall-mounted and ceiling-mounted cameras to mitigate baseline drift caused by longitudinal table movement [14]. In the calibration, a reflector block is manually placed sequentially at nine different points. While the central isocenter point can be precisely placed with the help of the internal lasers, manual placement of the block at the remaining eight points introduces uncertainties that propagate into the RGSC calibration, ultimately affecting the accuracy of patient motion management. In addition, baseline drift caused by couch sagging under patient weight can also be observed on the RGSC trace, limiting the accuracy of motion management during clinical applications. To resolve these clinical challenges, an enhanced calibration method was proposed, in which the calibration employed three marker blocks with the aid of lasers and CT console while a patient-equivalent load was present on the table.

## Materials and methods

Recently, we installed a wall-mounted RGSC camera paired with a Siemens go.Sim CT scanner. The RGSC system supports four modes: 4D scan, phase gating, amplitude gating, and breath-hold gating. All results presented in this paper are based on the breath-hold gating mode. Following the recommendations of AAPM TG 66 [20] and the vendor, a rigorous commissioning test was conducted for the go.Sim CT scanner. Sub-millimeter accuracy was confirmed for CT table movement and external laser alignment, while internal and external lasers demonstrated sub-millimeter-level coincidence.

### Varian calibration method

**Fig 1:**
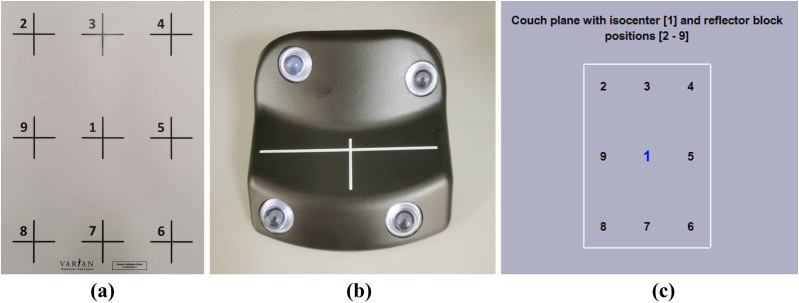
Varian calibration method. (a) Calibration plate; (b) Reflector block; (c) Calibration program interface.

Varian introduced a nine-point calibration method [14]. A calibration plate (Fig 1a), labeled with nine positions spaced 15 cm longitudinally and 10 cm laterally, was provided to facilitate the calibration procedure. In the calibration, a marker block (Fig 1b) was initially placed at the center of the plate and aligned with the CT isocenter with the help of internal lasers. The calibration program interface (Fig 1c) guided the process, wherein the marker block was sequentially positioned at points 1–9 on the plate, with images captured at each location. Upon completing the calibration, the marker block was repositioned at the isocenter for verification. The setup errors in all three directions were evaluated, and the verification was deemed successful if the errors remained below 0.5 cm.

### Proposed calibration method

**Fig 2:**
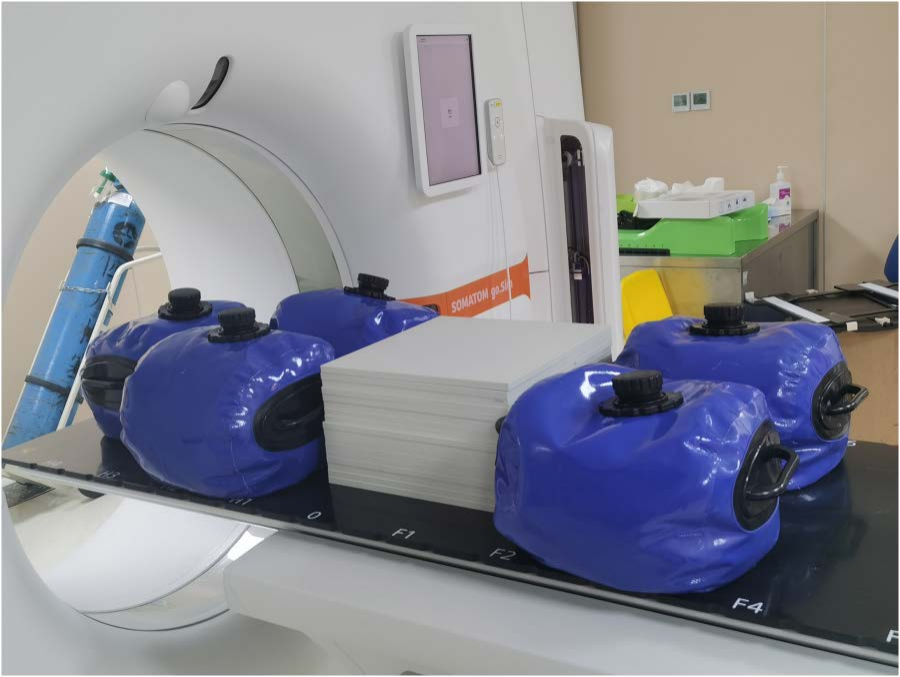
Distribution of the 70 kg weight.

**Fig 3:**
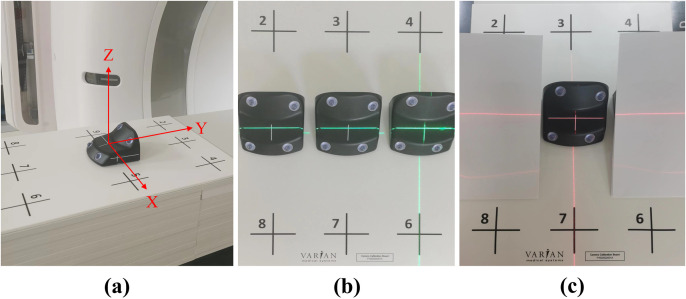
The proposed calibration method. (a) CT coordinate system; (b) Initial setup; (c) Calibration for position 1.

The proposed calibration method enhances the Varian calibration method through three major improvements:

(1) To eliminate baseline drift caused by couch sagging due to the patient’s weight during clinical applications, five bags filled with water and some solid water phantoms (approximately 70 kg in total) were distributed on the table (Fig 2).

(2) To remove human positioning uncertainties, lasers and couch movement were utilized to precisely position the marker blocks at all nine calibration points.
(3) To improve calibration efficiency, three blocks were employed and placed along a line with the help of external lasers, and the calibration was performed by moving the couch longitudinally (Y direction, as shown in Fig 3a) while shielding the other two blocks, instead of repositioning a single marker block across all nine points on the calibration plate.

The specific steps were as follows:

(1) The initial setup of the proposed method is shown in Fig 3b. A marker block was positioned at point 1 on the calibration plate and aligned with the external lasers, and the other two blocks were then placed at points 5 and 9 accurately with the assistance of the external lasers by shifting the ceiling light ±10 cm laterally (X direction, as shown in Fig 3a).
(2) The three blocks were moved to the internal laser plane by shifting the couch longitudinally from the console. Calibration for point 1 was then conducted by only exposing the block at point 1 to the camera’s view meanwhile manually occluding the other two blocks (Fig 3c). Similarly, calibrations for points 5 and 9 were performed by shielding the other two blocks during each calibration step.
(3) Through the console, the table was moved longitudinally +15 cm to calibrate points 2 ~ 4 and −15 cm to calibrate points 6 ~ 8. Marker blocks not at the calibration point were manually blocked at each step.

After calibration, verification was performed by shifting the couch back to the initial calibration position and only exposing the block at point 1 to the camera. Calibration would be successful if the block’s deviation in all three directions was less than 0.5 cm.

### Research method

In the physical coordinate system, it is relatively difficult to directly quantify the magnitude of the block positioning uncertainty. By quantifying the block position in the RGSC camera view coordinate system, the position uncertainty in the physical coordinate system can be reflected indirectly [19]. By repeating this measurement 10 times, the standard deviation of these camera view coordinates was calculated to estimate the positioning uncertainty.

To evaluate the residual drift signal after calibration, five bags filled with water and some solid water phantoms (approximately 70 kg in total) were placed on the CT table to simulate a patient, and a Varian breathing phantom with an attached marker block was placed on the solid water phantom to mimic the patient’s DIBH. The Varian breathing phantom features a rotating oval-shaped eccentric disc that drives a connected metal plate. After breathing pattern learning, the phantom was stopped at the maximum inhale position, and the DIBH scanning was initiated. For each calibration method, the scans under weight were repeated 15 times. For comparison, all scans were performed over a fixed 40 cm length starting from the same position. RGSC trajectories were exported in VXP format, and the noise was removed by smoothing the trajectory data with the moving average of 25 points that correspond to 1 s. Residual drift is obtained by calculating the difference between the maximum and minimum values of the smoothed data.

**Fig 4:**
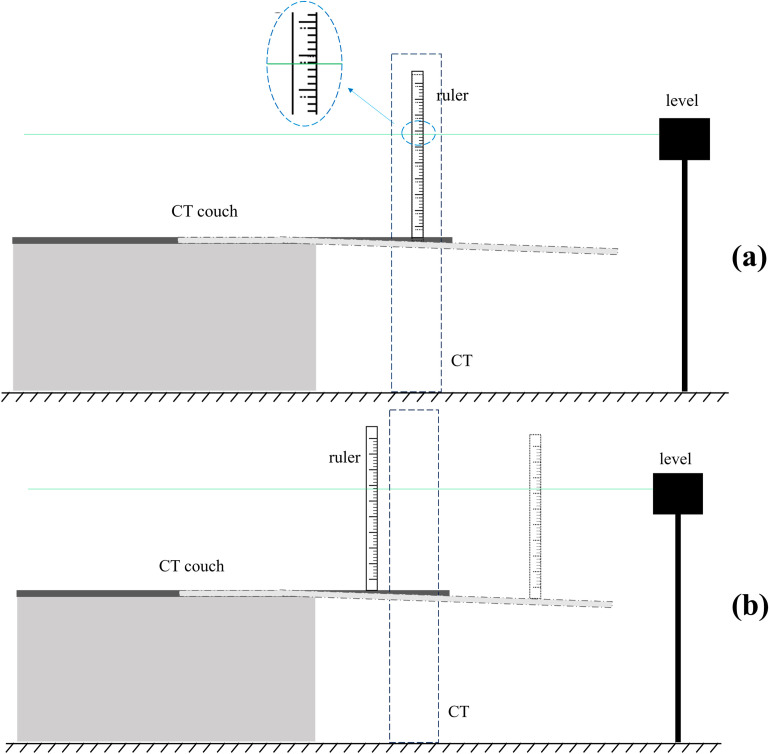
Schematic diagram of RGSC-like and CT-like measurements. **(a)** CT; **(b)** RGSC.

To compare the impact of CT table droop on RGSC signals and CT images, an experimental scheme was designed as illustrated in Fig 4. A ruler was secured to either the couch surface or the internal laser plane, depending on the simulation type. Measurements were taken every 5 cm as the bed moved longitudinally, up to a maximum distance of 130 cm.

SPSS 26.0 was used for statistical analysis. For all comparisons, independent samples t-test was conducted to verify whether the improvement was significant.

## Results

Table 1 tabulates the positioning uncertainties at points 2, 4, and 6, comparing the proposed method with the Varian calibration method. The proposed method significantly reduces uncertainties in the X and Y directions (p-value < 0.05). In contrast, uncertainties in the Z direction are negligible for both methods, since the calibration plate remains nearly level within a small area at each point and the reflector block exhibits almost identical Z coordinates at the exact location and its approximate locations for each point. In addition, for the proposed calibration method, the position uncertainty in the X direction is lower than that in the Y direction, which can be attributed to the structural design of the external laser system. This system comprises two sidewall lasers and one ceiling laser. The X position of the block is determined solely by the ceiling light, while the Y position is jointly determined by the two sidewall lights. Due to manufacturing limitations, the two sidewall lights cannot perfectly overlap, resulting in a broadened laser line and increased uncertainty in the Y direction.

**Table 1:** Coordinate uncertainty (standard deviation) comparison in camera view coordinate system, between Varian calibration method and the proposed calibration method.

	Lateral (cm)	Longitudinal (cm)	Vertical (cm)
**Point 2** ^**#**^	0.210	0.231	0.018
**Point 2** ^*^	0.008	0.057	0.012
**P-value**	0.040	<0.001	0.501
**Point 4** ^**#**^	0.233	0.236	0.023
**Point 4** ^*^	0.008	0.043	0.012
**P-value**	0.001	<0.001	0.148
**Point 6** ^**#**^	0.261	0.242	0.021
**Point 6** ^*^	0.010	0.023	0.009
**P-value**	0.002	<0.001	0.085

# The Varian calibration method.

*The proposed calibration method.

**Fig 5:**
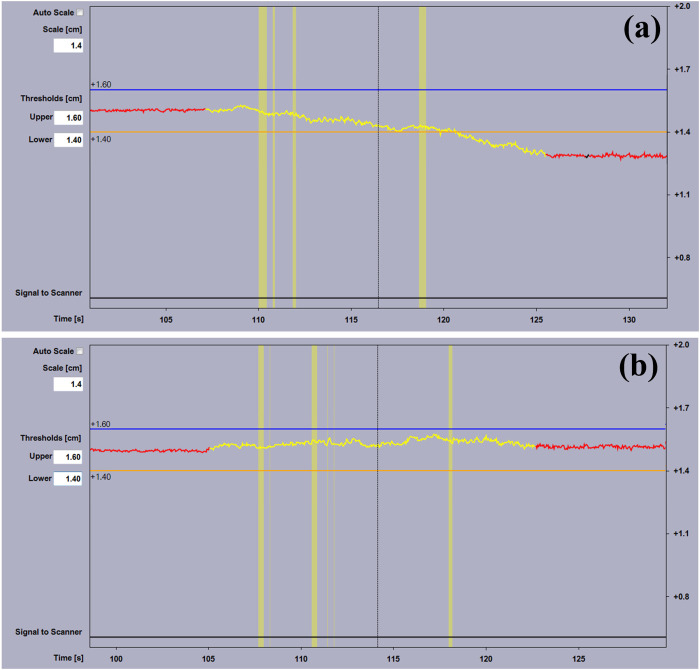
Comparison of different calibration methods. **(a)** Varian calibration method; **(b)** The proposed calibration method. The yellow segment indicates the CT beam-on time.

Fig 5 presents the RGSC trajectories for different calibration methods during the simulated DIBH scanning with approximately 70 kg of weight distributed on the couch. The scan length was fixed at 40 cm, and the couch was gradually moved away from the camera during the scanning. The trace for the Varian calibration method exhibits a downward drift of more than 2 mm as the table moves away from the camera, as depicted in Fig 5a. This drift is attributed to the rigid table support structure, which holds the inferior part of the couch while the superior part is suspended. As the couch moves forward, the unsupported portion increases, resulting in more severe sagging. This variability introduces more complexity to the DIBH scans. Such drift can potentially lead to the DIBH trace exceeding the predetermined thresholds during scanning, further resulting in inaccurate treatment. In contrast, the proposed calibration method demonstrates superior performance, effectively reducing the drift signal to less than 1 mm during the weight DIBH scan, as illustrated in Fig 5b. Table 2 summarizes the residual baseline drift for both methods. The residual baseline drift of the proposed calibration method is significantly lower than that of the Varian calibration method, and the difference is statistically significant (p-value < 0.001).

**Table 2:** Comparison of the residual drifts of simulated DIBH waveforms for Varian calibration method versus the proposed calibration method.

	Mean ± SD (mm)	Range (mm)
**Varian calibration method**	2.84 ± 0.22	2.42 ~ 3.25
**Proposed calibration method**	0.64 ± 0.06	0.57 ~ 0.77
**p-value**	<0.001	

Abbreviation: SD, standard deviation.

**Fig 6:**
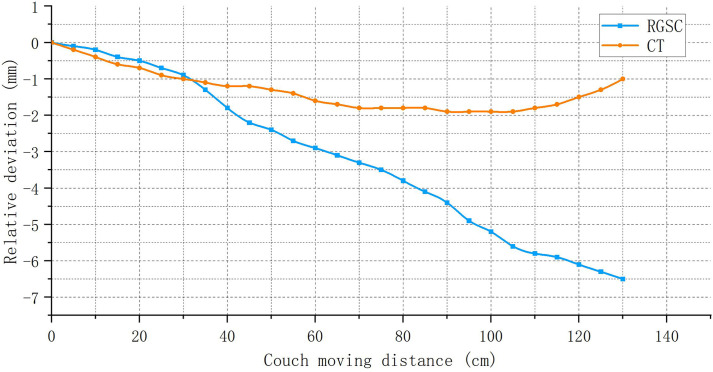
The influence of CT couch sagging on RGSC signal and CT image.

Fig 6 shows the effects of CT couch sagging on RGSC signals and CT images. The maximum downward displacement using CT-like measurement is within 2 mm, meeting the requirements for CT table flatness specified in AAPM TG 66 [20]. A trend is observed in the CT-like measurements where it initially sags and then rises, which may be attributed to the small spherical support structure at the front of the CT couch base. It is initially at a distance from the underside of the couch and does not provide support. As the couch moves outward and sags downward, the spherical support eventually touches the bottom of the couch, holding the couch in place. Using the RGSC-like measurement method, a maximum downward sagging of approximately 7 mm is observed, consistent with the results reported by Varian [17]. The result confirms that the baseline drift observed in Fig 5a is directly caused by CT couch sagging.

## Discussion

Respiratory-induced tumor motion poses a significant challenge in delivering precise radiotherapy, as it can lead to geometric errors that compromise tumor control and increase complications in surrounding healthy tissues [21]. To address this issue, various motion management strategies have been developed, including internal target volume (ITV) [22,23], mid-ventilation (MidV) [24,25], breath-hold (BH) [26,27], etc. These strategies rely on accurate motion management devices, such as RGSC, to enable 4DCT or breath-hold CT simulation. For RGSC systems with wall/ceiling-mounted cameras, respiratory signals are often contaminated by drift signals caused by CT couch sagging, introducing additional noise into respiratory waveforms. This noise can lead to inaccurate determination of DIBH thresholds, potentially resulting in suboptimal treatment planning and delivery. For 4DCT scans, it can cause inaccurate binning results, especially when using amplitude-based binning. Furthermore, when a visual coaching device (VCD) is used during CT simulation, it may mislead patients to alter their breathing patterns, further impacting treatment outcomes. To address this clinical challenge, an enhanced calibration method was proposed, reducing marker block positioning uncertainty to the sub-millimeter level and limiting baseline drift to within 1 mm. Significantly, although the method was tested with a wall-mounted camera, it is equally applicable to ceiling-mounted cameras, expanding its clinical utility.

The proposed calibration method was performed with a weight of 70 kg. It is important to note that residual drift signals may still occur for patients whose weight deviates significantly from this value, with the direction and magnitude of the drift contingent on the weight discrepancy. Nonetheless, the residual drift signal of the proposed method is expected to remain significantly smaller than that of the Varian calibration method under other patient weights. The impact of couch sagging due to patient weight can be further minimized if RGSC incorporates the ability to store multiple calibration results for various weight ranges, enabling users to select the most appropriate calibration for individual patients. We encourage vendors to consider incorporating this functionality in the next version of RGSC.

The proposed method fixes marker blocks to the CT table and then performs the calibration at different points by console-controlled table movement, ensuring a consistent and fixed relative position between the blocks and the CT table. This approach not only enhances calibration accuracy but also is more in line with actual clinical use. In contrast, the Varian calibration method requires repositioning the block, altering its relative position to the couch, and introducing additional uncertainties. RGSC’s ability to distinguish the position of the block in three-dimensional space makes both the center position and orientation of the block important during calibration. The use of lasers for marker block alignment is therefore strongly recommended. In addition, the proposed calibration method using laser-assisted and couch-shift positioning can get rid of the limitation of the physical calibration board provided by Varian.

Liu et al. compared baseline drifts on an empty couch using three reflector blocks versus a single reflector block in the calibration [19]. Through the help of setup laser and moving the couch during calibration, their three-block method effectively reduced the block positioning uncertainty and the baseline drift introduced by longitudinal relative movement between the block and the camera. Despite these improvements, we still observed a residual drift of 2.12 ± 0.19 mm during the weight DIBH scan. This limitation arises because the method aims to remove the baseline drift introduced by couch movement but does not account for couch sagging under patient weight.

**Fig 7:**
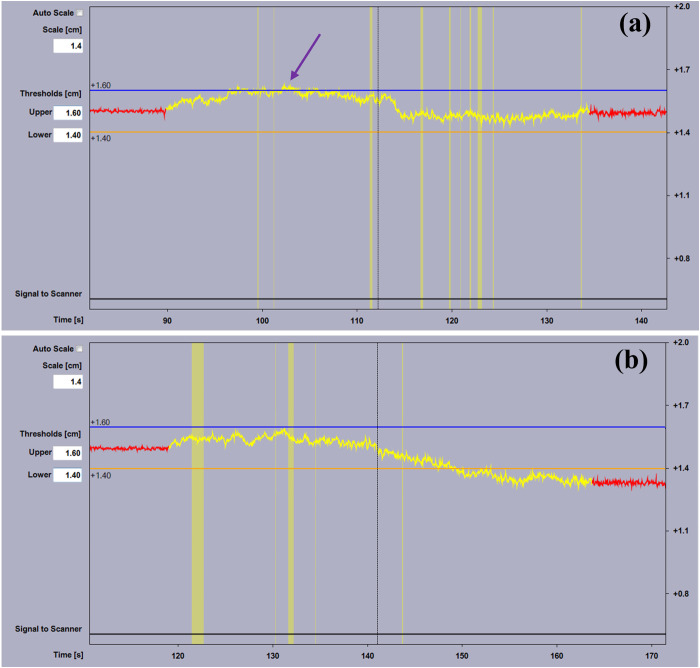
Comparison of two different calibration with weight for a scan length of 1 m. (a) The extended calibration proposed by Park et al. [18]; (b) The proposed calibration method. The yellow segment indicates the CT beam-on time. The purple arrow indicates the DIBH trace exceeding the predetermined thresholds during scanning.

A recent work conducted by Park et al. also took into account the weight factor and expanded the calibration range to improve baseline drift beyond the calibration area [18]. Our tests indicate that while it performs better drift correction for scans exceeding 1 meter, it was less effective for clinically typical scan lengths of 40 cm, as shown in Fig 7. It may even lead to the DIBH trace exceeding the predetermined thresholds during scanning due to over-calibration (as indicated by the purple arrow in Fig 7a). Moreover, this method relies on manual positioning, which introduces greater calibration uncertainty compared to the proposed method.

Shi et al. reported that RGSC systems achieved spatial accuracy within 1 mm in the X and Z directions over a range of 10 cm, but showed the worst accuracy of 2 mm in the Y direction, probably due to the camera’s less sensitivity to depth [15]. Our results are similar, except that the Y-direction accuracy observed in our RGSC system is slightly lower. This discrepancy may be attributed to differences in camera installation configurations since the camera is wall-mounted for us but couch-mounted for them. Further investigation is needed to evaluate the impact of camera installation on system performance in greater detail. Moreover, it is recommended to install the RGSC camera according to the manufacturer’s recommended range of 350–500 cm from the CT isocenter and the optimal projection angle. Our RGSC Camera is located in the optimal mounting area.

Although the proposed method demonstrates significant improvements in baseline drift, a more rigid couch appears to be a better solution if available. It not only eliminates baseline drift but also avoids the need for burdensome camera calibration, as the camera can be couch-mounted. Furthermore, a couch-mounted camera paired with a good rigid couch optimizes the clinical application of RGSC’s ability to identify the marker block in three-dimensional space. In contrast, the calibration method designed for wall/ceiling-mounted cameras can only reduce the baseline drift in the Z direction, but cannot alleviate drift signals in the other two directions. As a result, RGSC systems with these configurations can reliably recognize patient movement only in the Z direction during clinical applications.

## Conclusion

We proposed an enhanced calibration method for the wall-mount RGSC camera. Using laser-assisted alignment and couch-shift positioning, three marker blocks were employed for calibration while the couch was weight-bearing. The proposed method significantly reduced marker block positioning uncertainty and effectively eliminated baseline drift caused by CT couch sagging during DIBH and 4DCT scans.
